# Case Report: Multiple colorectal cancers in a patient with Ulcerative colitis and Lynch syndrome: Is there a role for prophylactic colectomy? A short report and review of literature

**DOI:** 10.3389/fonc.2022.1031606

**Published:** 2022-12-22

**Authors:** Adewale Adeoba Ayeni, Peter Waterland, Matthew Evans, Shika Singhal, Rajan Kumar Patel, Akinfemi Akingboye

**Affiliations:** ^1^ Department of General Surgery, The Dudley Group Foundation NHS Trust, Russells Hall Hospital, Dudley, West Midlands; ^2^ Department of Pathology, New Cross Hospital, Wolverhampton, United Kingdom

**Keywords:** HNPCC: hereditary non-polyposis colorectal cancer, IBD - inflammatory bowel disease, colorectal cancer, Lynch syndrome, Ulcerative colitis (colitis ulcerosa)

## Abstract

It is a known fact that Lynch syndrome (LS) and Ulcerative colitis (UC) are individually associated with increased risk of colorectal cancer. While there is no conclusive evidence to demonstrate a cumulative risk when these two conditions coexist, available data suggest early onset and synchronous cancers are synonymous to this group. We have reported an unusual case of multiple synchronous colorectal cancers in a young man with ulcerative colitis and Lynch syndrome also known as Hereditary Nonpolyposis Colorectal Cancer (HNPCC) gene mutation. We propose that conducting a detailed genetic mutation profile in LS patients may play a key role in guiding the intensity of endoscopic surveillance and that a concerted, pragmatic, patient guided approach should be adopted on the subject of prophylactic colectomy when UC and LS co-exist.

## Background

The development of colorectal cancer remains one of the most dreaded consequences of ulcerative colitis (UC) (1). Colonic lesions in these cases are frequently multiple and tend to occur in younger patients. Colorectal cancer (CRC) on a background of ulcerative colitis develops through a sequence of mucosal transformation from chronic inflammation to dysplasia and eventually to invasive adenocarcinoma (2). Therefore, colonoscopic surveillance is recommended with the aim of early detection of precancerous or cancerous lesions in-order to reduce the risk of mortality from colorectal cancer in these patients. Ulcerative colitis is associated with a 1–2% risk for CRC after 10 years of disease, and this risk increases by 0.5–1% annually thereafter (3).The risk of developing colorectal cancer in Inflammatory bowel disease (IBD) is about 1.7 times greater than that of general population (4).

Lynch syndrome (LS) is known to carry the most common inherited colorectal cancer predisposition (5). The predisposition results from deficient mismatch repair gene that has compromised the ability to repair base-pair mismatches in Deoxyribonucleic acid (DNA). It is characterized by predominance of right sided colon cancer with a propensity for synchronous and metachronous lesions (5, 6). Although, CRC is the most common cancer associated with LS, cancers of the small bowel, endometrium, ureter, renal pelvis, hepatobiliary, ovary and stomach can also occur (5). LS accounts for about 3% of all new cases of colorectal cancer (5, 7). It is associated with a 6–77% lifetime risk for CRC that is determined in part by the particular mismatch repair (MMR) protein gene mutation. This reported lifetime CRC risk estimates range from 25 to 70% for MLH1 mutation carriers, 25 to 77% for MSH2 mutation carriers (8, 9), 6 to 22% for MSH6 mutation carriers (8, 10), and 8 to 34% for PMS2 mutation carriers (11). EPCAM mutation carriers have a risk comparable to MSH2 mutation carriers (12).

CRC has been shown to occur at a younger age in patients with concomitant LS and Inflammatory bowel disease (IBD) (4). Theoretically, this could suggest a cumulative risk in these patients, although there is not enough scientific evidence to demonstrate this. Limited literature (**Table 1**) on this group of patients makes the formulation of surveillance guidance or recommendation of prophylactic treatment options difficult. Detailed analysis of genetic molecular markers in these cases may help to individualize treatment and support use of advance endoscopy for surveillance and selection of patients who may benefit from prophylactic colectomy. This case report illustrates the role of genetically guided and individualized surgico-oncological treatment for patients with concurrent LS and UC. It further emphasizes the need for targeted and tailored colorectal surveillance program with anticipatory approach in managing colorectal cancer complexity and recurrence.

**Table 1:** Literature review of CRC in patients with concurrent IBD and LS (Lynch syndrome).

Authors	Age	LS mutation	Cancer type	Stage	Location	Treatment
Matsuda et al., 1999 (13)	–	Amsterdam criteria diagnosed	Non-synchronous	–	Rectum	Resection of rectal stump
Minami et al., 2014 (14)	28	MSH2	Non-synchronous	–	Hepatic flexure	Total proctocolectomy
McNamara et al., 2015 (15)	46	MLH1	Non-synchronous	–	–	Colectomy
	33	PMS2	Non-synchronous	–	–	Colectomy
Derikx et al., 2016 (4)	34 37	MLH1 MLH1	3 synchronous CRC 3 synchronous CRC	T2N1Mx (III) T4aN2M1(IV)	Rectum Sigmoid and appendix	Excision of rectum and os coccyges amputation, neoadjuvant chemoradiation Total colectomy, adjuvant chemotherapy, HIPEC
	34	MLH1	3 synchronous CRC	T2N0M0 (I)	Sigmoid	Subtotal colectomy
	38	PMS2	Non-synchronous	TxNxM1 (IV)	Hepatic flexure	–
	42	MSH6	Non-synchronous	T3N2M0 (III)	Splenic flexure	Left hemicolectomy, adjuvant chemotherapy
Barberio et al., 2022 (16)	66	MSH2	–	–	Sigmoid	Sigmoid resection
	28	MSH2	–	–	–	Ileal pouch-anal anastomosis
	49	MSH2	–	–	–	Ileostomy
	59	MSH2	–	–	–	Ileostomy
	47	MLH1	–	–	–	Ileostomy
	56	MLH1	–	–	Left colon	Left hemicolectomy
	35	MSH6	–	Stage IV	CRC with metastasis	–

## Case presentation

**Figure 1:**
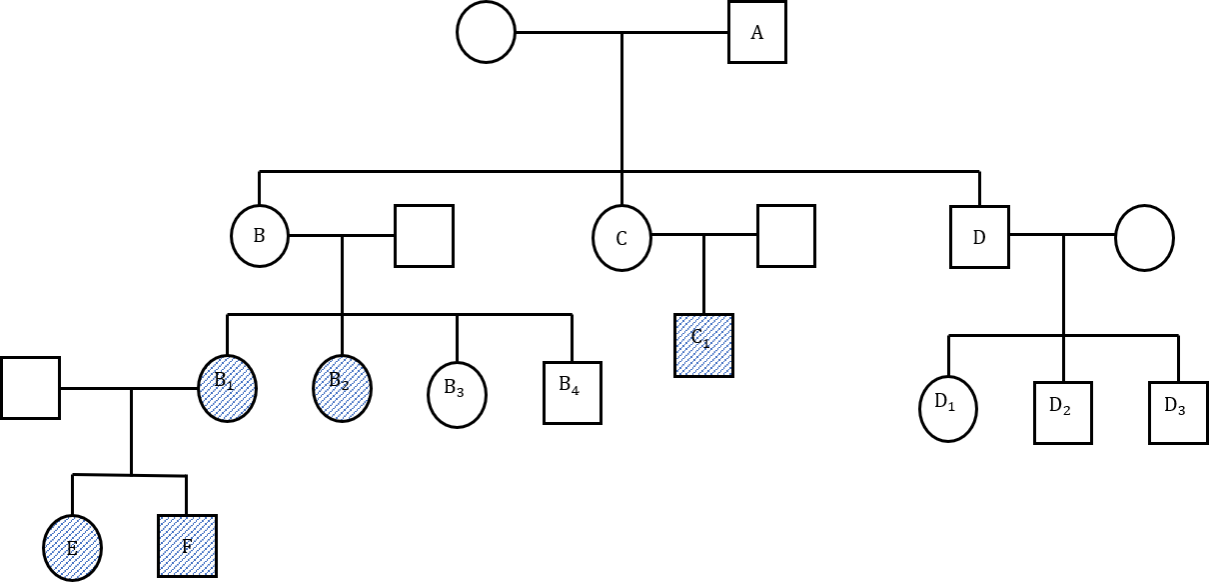
(Family pedigree). A: Bowel cancer at 54 years, not tested for lynch syndrome (LS) gene. B: Rectal cancer at 33 years, uterine cancer at 50 years, pancreatic cancer at 78 years, not tested. B1: hMSH2, colon cancer at 34 years. B2: MLH, PMS2, endometrial cancer and intraductal pancreatic neoplasia at 62 years. B3: LS gene negative. B4: LS gene negative. C: Renal cancer in 30s, not tested. C1: Bowel and kidney cancer at 30 years, LS gene positive. D: Bowel and liver cancer at 75 years. D1: LS gene negative. D2: LS gene negative. D3: LS gene negative. E: MSH2, Colon cancer at 32 years (Index patient). F: MSH2.

A 32 years-old male diagnosed with UC 7 years prior presented with non-bloody loose stool about 8-12 episodes per day and anemia with no extra-intestinal manifestations. He was also known to have MSH2 mutation following genetic testing due to a strong family history of LS as depicted in the pedigree (**Figure 1**).

**Figure 2:**
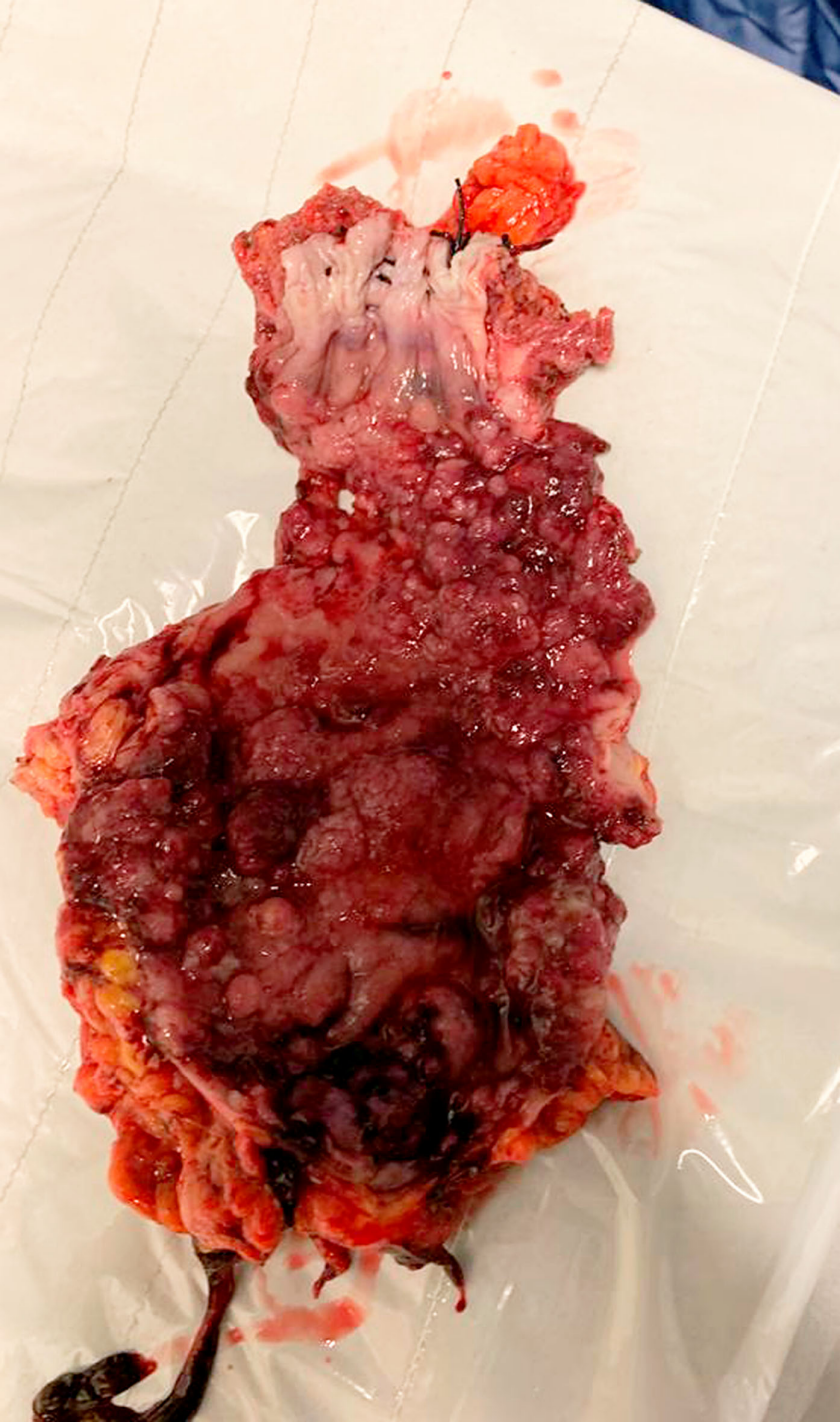
Gross specimen showing multiple foci of adenocarcinoma on background of tubulovillous adenoma with dysplasia in rectum.

He had been managed conservatively using mesalazine and steroids until 2 years prior to presentation when he had an open right hemicolectomy for caecal low grade dysplastic tubulo-villous adenoma found on surveillance colonoscopy. He had one surveillance colonoscopy performed a year after his initial procedure with findings of active colitis and single low-grade tubulovillous adenomas in the colon and rectosigmoid junction. Colonoscopy and biopsy done after index presentation revealed moderately active colitis, multiple rectal and sigmoid tubular adenomas with low grade dysplasia. An elective laparoscopic completion proctocolectomy and ileoanal pouch was proposed. However, intraoperative findings of left pelvic wall abscess with grossly thickened sigmoid colon mass coupled with extensive disease of rectum down to anorectal junction (**Figure 2**) necessitated an open completion proctocolectomy with terminal ileostomy.

Histology from the specimen revealed moderately differentiated adenocarcinoma arising from tubulovillous adenoma. The whole large bowel mucosa showed extensive adenomatous transformation with areas of low and high-grade dysplasia. Multiple foci (at least 21) of carcinomatous transformation were seen within these adenomas (pT3pN1bpMx, Dukes stage: C1, R0). We performed microsatellite instability (MSI) testing on sections from FFPE (formalin-fixed paraffin embedded) tumor blocks using the Idylla MSI Test. All four tumors tested were MSI-H. This is suggestive of an underlying mismatch repair defect (MMR), and further testing for MMR protein expression was performed using the VENTANA MMR antibodies. The adenomatous component showed preserved expression of MSH2 and MSH6, while the invasive component showed loss, consistent of Lynch syndrome. Next-generation sequencing (NGS) was performed using the pan cancer TruSight Tumor 170 kit on the NextSeq platform, KRAS and MSH2 gene mutation were detected from both the tubulovillious adenoma and the invasive carcinoma samples. A PIK3CA mutation was also detected in the invasive component. They were both negative for NTRK fusions. NRAS, BRAF, EGFR, APC and CTNNB1 mutation testing failed.

**Figure 3:**
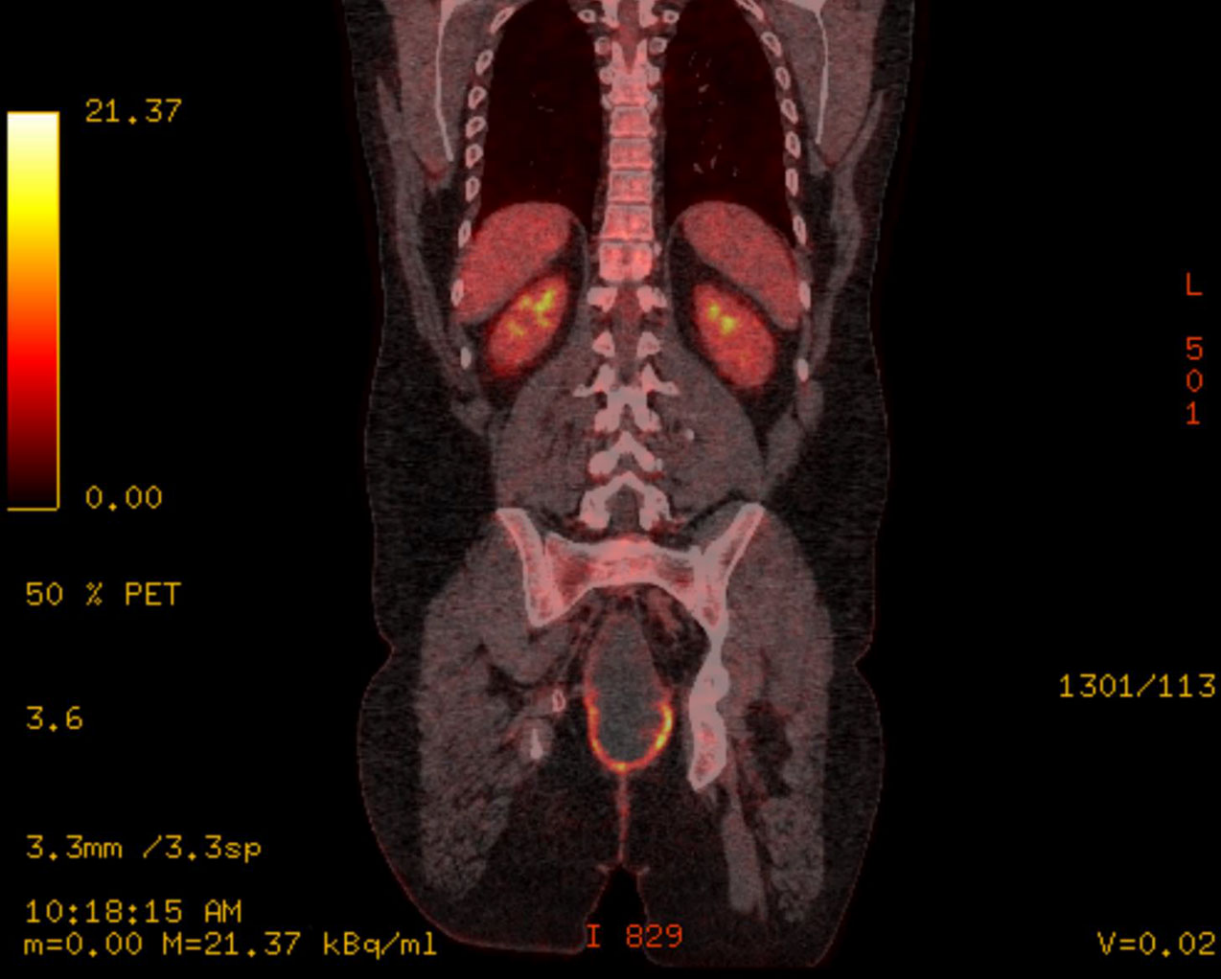
PET scan showing pelvic cystic lesion.

Following surgery, he had adjuvant chemotherapy with capecitabine and oxaliplatin (8 cycles) followed up by the standard colorectal surveillance program. During his second year of follow up, he developed some mucoid discharge from a perineal sinus. Further imaging computer tomography (CT) scan then Positron emission Tomography (PET) Scan (**Figure 3**) revealed a pelvic cystic lesion, which was inconclusive on the PET scan.

Biopsy taken form the discharging sinus showed features of dysplastic glands in keeping with metastatic adenocarcinoma of colorectal origin. These findings were discussed at the complex colorectal cancer multidisciplinary team meeting and outcome was discussed with the patient which was either to commence palliative immunotherapy (Pembrolizumab) or to undergo extensive radical total pelvic exenteration. He opted for palliative immunotherapy which he has commenced.

## Discussion

Our patient had endoscopically irresectable polypoid tubulovillious adenomas in the caecum for which he underwent right hemicolectomy with subsequent endoscopic surveillance. Two years later, he was found to have multiple low grade dysplastic polypoid lesions in the rectosigmoid area with background active ulcerative colitis. He underwent completion proctocolectomy due to the extensive nature of the polypoid lesion and encroachment of the anorectal junction but developed pelvic recurrence 18 months following adjuvant chemotherapy. We present the first case of multifocal (at least 21) synchronous adenocarcinoma of the sigmoid and rectum confirmed on histology in a patient with history of both LS and UC.

According to Bonadona et al, our patient’s estimated cumulative risk of CRC (based on his LS genetic profile) by 70 years of age is 48% (95% CI, 30% - 70%) while Dowty et al. puts this estimate at 34% (8, 9). Thus, CRC risk varies in patients with MSH2 mutation. Although 17% of males with MSH2 mutations have estimated CRC risk of 0 – 10%, the risk of CRC is up to 100% in 18 percent of MSH positive men. As for UC, it is associated with up to 5% of all colorectal cancers. Several factors including male gender, young age at diagnosis and presence of extensive disease have been associated with the increase risk of CRC in UC patients (9). Therefore, presence of concomitant UC in LS could further increase his risk of CRC.

The chronic mucosal inflammation in UC induces a field change of cancer-associated molecular alterations resulting in epithelial dysplasia. CRC in UC is hypothesized to develop from dysplasia (4, 17). As a result of this mechanism, surveillance colonoscopy programs have been advised in order to reduce the CRC-associated morbidity and mortality in UC (18). According to the British, European and American endoscopy guidelines, surveillance endoscopy for dysplasia in IBD should begin between 6 to 10 years following diagnosis. Colonoscopy surveillance have shown promising result by reducing the risk of colonic cancer incidence and earlier detection stage of colonic cancer lesions as evidenced by Cochrane review of case controls and the St Mark’s program (19).

The 2015 SCENIC consensus strongly recommends chromoendoscopy or high-definition white-light endoscopy for detection of dysplasia on surveillance in UC patients, in addition to colonoscopically targeted biopsies over random biopsies with aim of improving outcome. The British society of gastroenterology, European society of gastrointestinal endoscopy (ESGE) and American gastroenterological association have all recommended surveillance colonoscopy in patients with Lynch syndrome with the aim of identifying and removing premalignant lesions and reduction of cancer associated mortality by early detection of malignant lesions (17, 20). A recent (2020) well-designed, adequately powered randomized parallel trial in Lynch syndrome patients found that results from high-definition white-light endoscopy was comparable to pan-colonic chromoendoscopy if performed by experienced and dedicated endoscopist (21).

Despite these advances, CRC risk in LS is not completely eliminated by colonoscopic surveillance. In fact, the finding of mismatch repair deficient crypt foci in apparently normal colon, have led to the suggestion that there may be an “invisible pathway” to CRC without an endoscopically detectable polyp precursor and the lifetime risk of CRC in patients with high-risk genotypes (MLH1, MSH2) on colonoscopy surveillance have been found to be around 40 percent (22, 23). Caruso et al. offered a genetic hypothesis that proposed a suppressive effect of each condition on the other (24). Authors proposed that clinical IBD requires both susceptibility and development genes. They postulated that mutations causing LS suppress the IBD development genes such that LS patients develop only a subclinical asymptomatic form of IBD.

More recently, two large pathology reviews attempted to better characterize the pathogenesis of IBD-associated CRC by examining histomorphologic features of these cancers in comparison with the histomorphology of microsatellite-stable (MSS) CRC, sporadic microsatellite instability-high (MSI-H) CRC, and LS-related CRC (22, 23, 25). Both groups demonstrate that IBD-associated CRC has morphologic similarities to both sporadic and hereditary MSI-H CRC compared to sporadic MSS CRC but also some unique features. Svrcek and colleagues further compared MSI-H and MSS IBD-associated CRC and show that MSI-H IBD-associated CRC even more closely resemble other MSI-H CRC. Taken together, the results suggest that IBD-associated CRC and LS-related CRC may share some common molecular events, but important differences in carcinogenesis generate the unique histomorphology of IBD-associated CRC. Furthermore, Svrcek et al. report a low frequency of MLH1 promoter methylation in IBD-associated CRC and suggest there are alternative mechanisms of MMR deficiency in these tumors. Later work by this same group proposes that dysregulation of inflammation-related micro-RNAs targeting MMR proteins may have a role in IBD-associated CRC development (26). The interrelation of CRC predisposition in LS and IBD may become clearer as more is understood about the pathogenesis of IBD and IBD-associated CRC, immune dysregulation in CRC, and the factors the modify cancer risk in LS patients.

Prophylactic colectomy is not considered a standard or necessary intervention for primary colorectal cancer risk reduction in patients with UC or LS. This may be because of the efficacy of colonoscopy surveillance in either group of patients. However, as available evidence suggests early onset of CRC in patients who have both LS and UC and the inherent propensity for synchronous CRC associated with this group. Total colectomy could serve both therapeutic and prophylactic purpose in patients who have developed CRC requiring segmental colectomy, although there is currently no proven survival benefit for more extensive surgery

Next generation sequencing based analysis has thoroughly characterized MSI positive cancers. MSI is a valuable diagnostic marker of LS and a potential predictive marker for response to immunotherapy and chemotherapy. This has made MSI and MSI- associated molecular changes in tumor of significant clinical importance with diagnostic and therapeutic implications (27).

Considering his background of both UC and LS, we propose that our index patient and other patients with similar background (concurrent IBD and LS) who develop CRC may benefit from panproctocolectomy in the first instance or a restorative proctocolecctomy rather than total colectomy or segmental resection. This decision could be better guided by the genetic mutation profile, lifetime risk of cancer using next generation sequencing. Furthermore, a more intense surveillance protocol that is personalized would perhaps help in detection of early recurrence in patient with MSI-High and deficient MMR, KRAS, NRAS and BRAF wild type. The use of monoclonal antibodies with specific targets has shown efficacy for advanced metastatic colorectal cancer, this principle has recently been explored in the treatment of colorectal cancer from lynch syndrome and ulcerative colitis with some promising results.

## Conclusion

Patients with concurrent UC and LS have a greater CRC risk hence surgical management of such patients should be individualized. Available evidence suggests that these patients have a much higher risk of developing synchronous colorectal cancer at a younger age. In addition to the findings on colonoscopy surveillance, further genetic testing is required in making the decision for surgical-oncological treatment before intervention. We therefore propose that advanced colonoscopy is preferred to standard endoscopy for surveillance, with low threshold to offering panproctocolectomy as the first treatment option. Furthermore, we recommend a formal national registry that would capture the various management strategies which could be used to produce consensus for such rare conditions.

## Data availability statement

The original contributions presented in the study are included in the article; further inquiries can be directed to the corresponding author.

## Author contributions

AAA: Write up of manuscript and literature review. PW: Patient recruitment and contribution to patient management. ME: Molecular pathologist who performed and interpreted genetic mutations. SS: Histopathologist who provided the histopatholgy slides and annotation of the slides. RP: Operating surgeon who provided gross specimen images. AA: Study design, conceptualization of study and overall supervisor of study (Senior Author). All authors contributed to the article and approved the submitted version.
